# Design and Evaluation of Paeonol-Loaded Liposomes in Thermoreversible Gels for Atopic Dermatitis

**DOI:** 10.3390/gels9030198

**Published:** 2023-03-05

**Authors:** Yu Wang, Yan Yue, Ruoyang Jia, Xinyi Liu, Zhiqing Cheng, Yongfeng Cheng, Yinxiang Xu, Zili Xie, Hongmei Xia

**Affiliations:** 1 College of Pharmacy, Anhui University of Chinese Medicine, Hefei 230012, China; 2 Clinical College of Anhui Medical University, Hefei 230031, China; 3 School of Life Science, University of Science and Technology of China, Hefei 230027, China; 4 Zhaoke (Hefei) Pharmaceutical Co., Ltd., Hefei 230088, China; 5 Anhui Institute for Food and Drug Control, Hefei 230051, China

**Keywords:** paeonol, thermosensitive gel, liposome–hydrogel, antioxidant, atopic dermatitis

## Abstract

Paeonol (PAE) is a hydrophobic drug. In this study, we encapsulated paeonol in a lipid bilayer of liposomes (PAE-L), which delayed drug release and increased drug solubility. When PAE-L was dispersed in gels (PAE-L-G) based on a poloxamer matrix material for local transdermal delivery, we observed amphiphilicity, reversible thermal responsiveness, and micellar self-assembly behavior. These gels can be used for atopic dermatitis (AD), an inflammatory skin disease, to change the surface temperature of the skin. In this study, we prepared PAE-L-G at an appropriate temperature for the treatment of AD. We then assessed the gel’s relevant physicochemical properties, in vitro cumulative drug release, and antioxidant properties. We found that PAE-loaded liposomes could be designed to increase the drug effect of thermoreversible gels. At 32 °C, PAE-L-G could change from solution state to gelatinous state at 31.70 ± 0.42 s, while the viscosity was 136.98 ± 0.78 MPa.S and the free radical scavenging rates on DPPH and H_2_O_2_ were 92.24 ± 5.57% and 92.12 ± 2.71%, respectively. Drug release across the extracorporeal dialysis membrane reached 41.76 ± 3.78%. In AD-like mice, PAE-L-G could also relieve skin damage by the 12th day. In summary, PAE-L-G could play an antioxidant role and relieve inflammation caused by oxidative stress in AD.

## 1. Introduction

Atopic dermatitis (AD), also known as atopic eczema, is a chronic relapsing inflammatory disease that is often associated with other atopic conditions such as asthma, rhinitis, and food allergies [1,2]. The main manifestations are skin barrier dysfunction, skin itching, etc. [3,4,5]. Its onset is usually concentrated in early childhood, but 2–10% of adults have AD, leading to a significant decrease in quality of life and increased psychological burdens [6,7,8,9]. The factors causing AD are not clear at present, but are mainly attributed to filaggrin (FLG), resulting in impaired skin barrier function [10] and changes in immune system function [11,12]. There are also possible environmental, genetic, and other factors. Currently, local corticosteroid drugs are used to treat moderate to severe AD patients clinically, but long-term use will lead to skin thinning, epidermal atrophy, vascular dilation, and other adverse reactions [13,14], and can even lead to patients’ poor compliance with the drugs. Therefore, to develop a natural medicine as an alternative therapy, Komal [15] took inspiration from curcumin and encapsulated tetrahydrocoumarin in a solid lipid nanoparticle gel, which enhanced the targeting of the drug to the skin in a controlled release manner, overcoming the skin barrier and improving bioavailability. Gallic acid, which has been shown to be effective for AD treatment, can be combined with a pH/temperature responsive gel for enhanced transdermal delivery [16]. Therefore, using Chinese herbal ingredients to treat AD has become a new trend.

Paeonol (PAE) is an active ingredient that is isolated from the dried roots or whole grass of *Cynanchum paniculatum* (Bunge) *Kitagawa, Paeonia fruticosa Andr,* and *P. lacti flora Pall* in the Ranunculaceae family [17]. A large number of studies have shown that paeonol has good anti-inflammatory [18], anti-allergy [19], anti-infection [20], and antioxidant [21] effects. However, paeonol has poor solubility in water, is volatile at room temperature, and has poor bioavailability, and so it is necessary to design smart polymer materials to deliver the drug. In addition to their ability to encapsulate hydrophilic and hydrophobic drugs, liposomes also provide better permeability and locally increased drug concentration, thereby reducing the stimulation potential [22]. Liposomes are an effective drug delivery system for the treatment of skin inflammatory diseases, because compared with traditional preparations, liposomes can carry a higher amount of drug to the affected area [23]. However, liposomes have poor stability, so they are modified in a variety of ways to increase it.

Hydrogels have been widely used in drug delivery, regenerative medicine, cosmetic technology, and other fields. Due to their three-dimensional network structure, combined with weak cohesion in the form of cross-linked covalent bonds and hydrogen or ionic bonds [24], hydrogels can swell in aqueous solution and have high water absorption. Since the human body is composed of a large amount of water, hydrogel polymers can penetrate deep into the skin [24]. In cosmetic applications, hydrogels are mainly applied to the scalp and skin and used in oral care [25,26]. In recent years, it has been found that these hydrogels, as transport carriers of natural plants, are beneficial for promoting hair growth [27] and protecting the scalp via pH value changes [28]. In the treatment of atopic dermatitis, damage to the skin barrier caused by inflammatory symptoms leads to increased cuticle transepidermal water loss, and it has been shown that the skin epidermal barrier and homeostasis are affected by age [29]. Hydrogels are breathable and moisturizing [30,31,32] and they reduce water loss, alleviate dry skin symptoms, and maintain skin elasticity. As a new type of topical preparation that has emerged in recent years, temperature-sensitive gels have the advantages of easy application and good biocompatibility. Poloxamers are ABA-type triblock copolymers consisting of PEO (A) and PPO (B) units. Poloxamer 407 (P407) requires a lower concentration for gel formation at 25 °C than other poloxamer series. At room temperature (25 °C), the solution appears as a flowing viscous liquid; at the gelling temperature, it changes into a semi-solid transparent gel [33,34]. At temperatures above the low-critical solution temperature, poloxamer will lose its PPO block, resulting in micellar formation and solution conversion to gels. Unfortunately, the low gelation temperature of poloxamer 407 (P407) as an in situ gel carrier was reported to impede drug administration [35]. Therefore, the addition of poloxamer 188 (P188) as a polymer additive can reduce prescription dosage and achieve heat sensitivity to the physiological environment. Poloxamer can also be used as a surfactant [36], emulsifier, and slow-release material [37,38]. It is easily absorbed after local administration and has good stability. However, poloxamers can form a hydrogel containing a large amount of water, which can cause a blasting reaction such that drugs are released in large quantities in the initial stage [39], preventing drugs from having a long-term therapeutic effect.

Liposome-in-gel systems combine the advantages of liposomes and gels; liposomes can reduce the explosive release of hydrogels [40], and gels can maintain the integrity and function of liposomes, thus improving the bioavailability of drugs [41]. Liposomes in thermosensitive gels have previously been shown to achieve slow-release functionality [42,43].

In this study, paeonol-loaded liposomes were prepared using the thin-film dispersion method. P407 and P188 were used to design heat-sensitive in situ gels to achieve the double delivery of a paeonol-loaded liposome-in-gel for transdermal drug delivery [44]. We produced the solution at room temperature, which can rapidly undergo phase transformation to form gels after being applied to the inflammatory site (32–37 °C), to prolong the retention time of PAE on the skin surface and increase the drug action time, and to provide a platform for the development of transdermal drug delivery. In this study, we investigated the antioxidant capacity of paeonol solution, paeonol-loaded liposomes (PAE-L), paeonol-loaded gels (PAE-G), paeonol-loaded liposomes in temperature-sensitive gels (PAE-L-G), and their effect on AD-like skin of mice induced by 2,4-dinitrochlorobenzene (DNCB).

## 2. Results and Discussion

### 2.1. Standard Curve of Paeonol

We selected the corresponding concentration value in the appropriate range (0–0.015 mg/mL) to draw the standard curve and take the concentration–absorbance as the coordinates. The linear regression equation is written as follows: A = 85.126 × C + 0.0117, and the regression coefficient was R^2^ = 0.9992, indicating that the linearity was good in this concentration range.

### 2.2. Characterization of PAE-Loaded Liposomes

Because liposomes are composed of a kind of nanoscale artificial vesicle and consist of phospholipids and cholesterol, PAE-L appears to the naked eye as a thick, yellowish liquid. The shape of PAE-L under an electron microscope (Glacios-200 KV, Thermo Fisher Scientific, Waltham, MA, USA) appears to be spherical. The hydration particle sizes of B-L and PAE-L were 127 ± 8.1 nm and 132.6 ± 11.5 nm, respectively. Zeta potential was negative (−19.4 ± 0.8 mV and −17.9 ± 2.1 mV). The encapsulation rate of PAE-L was 86.47 ± 8.22%.

### 2.3. Physicochemical Characterization of Formulations

The surface temperature of the skin was 32–35 °C [45]. When the body suffers from skin damage, its temperature will slightly rise by 0.5–1 °C [46]. Therefore, when the temperature-sensitive in situ gels act on the skin surface, the minimum gelling temperature is 32.5–34 °C, and the maximum is 36–37 °C. Therefore, the gelling temperature of the prescriptions should be between 32 °C and 37 °C. In order to obtain the most suitable carrier for the skin surface of AD mice, poloxamer gels (Table 1), which are temperature-sensitive at 32 °C, 35 °C, and 37 °C, respectively, were selected to check their physical and chemical properties. In the case of the same polymers and similar concentration compositions, both PAE and PAE-L have less influence on the gelling temperature than the blank in situ gels. For temperature-sensitive gels, it is necessary to quickly change from solution to gel form in a short time. Although the gelling ability of PAE-L-G was weakened at 32 °C, 35 °C, and 37 °C, requiring 51.80 ± 5.05 s, 62.92 ± 7.67 s, and 82.58 ± 2.34 s, respectively, there was no significant difference compared to blank gels. In addition, the gelling temperature and gelling capacity were mainly affected by the prescription amounts of P407 and P188 in the gels. The viscosity had been reported to be affected by increasing polymer concentration or use [47,48]. Therefore, viscosity levels need to be within the appropriate range of values. There was no significant difference between the viscosity of blank gels and PAE-G, but the viscosity of the formula was significantly improved after the addition of liposomes, and the viscosity could even be higher than 100 MPa.S at the gelling temperature. The ratio of prescription dosage had little influence on the viscosity change.

In this study, temperature-responsive gels were prepared using the cold method. The solutions of gels were seen clearly by visual observation under black background. The pH of the skin surface was first determined by Heuss in 1892 [49]. The main reason for this is that both the lipid tissue and acidic environment can provide better conditions for lipid metabolism. Studies have shown that the pH level rises in patients with atopic dermatitis [50]. Therefore, the pH value of the skin carrier is an important parameter because differentiation of the skin pH can regulate adverse reactions such as redness or itching [51]. As can be seen from the pH data in Table 1, the pH values of blank gels were measured as 7.04 ± 0.02, 7.11 ± 0.12, and 7.27 ± 0.17 at the gelling temperature. For PAE-L-G, the pH values were 7.18 ± 0.03, 7.23 ± 0.01, and 7.38 ± 0.14, and the pH value decreased with the increase in temperature. The dosage of prescription and the loading of PAE had little effect on the pH value. In addition, skin carriers with alkaline had been reported to improve skin health in some conditions [52]. Therefore, the medicinal carrier in this study can play a role in the treatment of atopic dermatitis.

Combined with the above results, it can be seen that although the temperature-sensitive gels can form semi-solid medicinal carriers on AD-like skin within the range of 32–37 °C, there is no significant difference, and the encapsulated PAE or PAE-L-G would not have a great impact on their physical and chemical properties. However, at the gelling temperature of 32 °C, the concentration of P407 and P188 required for prescription is small, which can reduce the cost of medicine and achieve the ideal therapeutic effect. Therefore, the thermosensitive gels at 32 °C were selected for the follow-up study on AD inflammation diseases.

### 2.4. In Vitro Permeation Study

**Figure 1:**
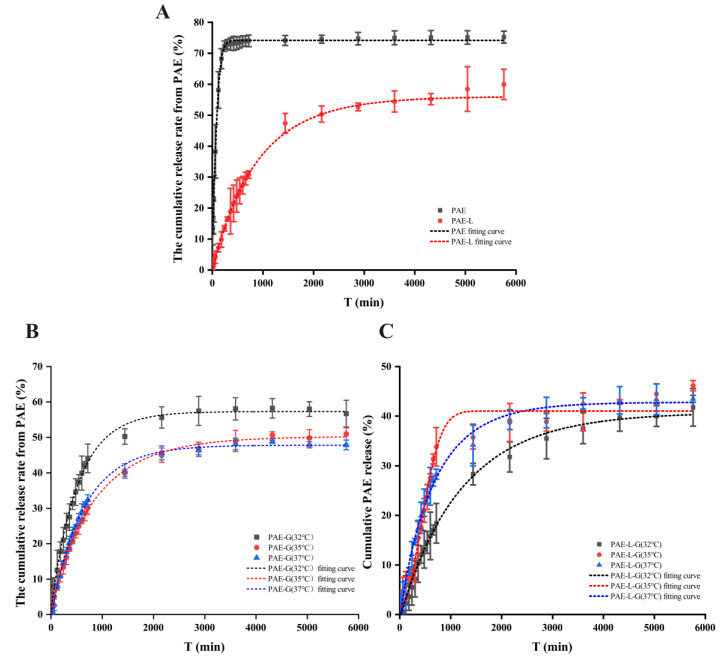
WeibullCDF fitting curve of different formulations. (**A**) PAE and PAE-L. (**B**) PAE-G at 32 °C, 35 °C, and 37 °C. (**C**) PAE-L-G at 32 °C, 35 °C, and 37 °C.

PAE is a lipophilic drug, but its solubility and skin accumulation can be improved by the carrier of liposomes and gels. According to the analysis of the dialysis release curve (Figure 1A), the cumulative release rates of the PAE group were 72.32 ± 1.68% within 12 h, while the maximum cumulative release rate of the PAE-L group was only 59.94 ± 4.88%. Therefore, the PAE solution can release the drug quickly in a short time, while the liposome group plays a sustained-release role. As Figure 1B,C show, PAE-L and PAE-L-G could achieve their effect in the local long-term due to sustained and controlled release in the treatment process. Among them, the cumulative release rate (R%) from PAE-L-G was the lowest, mainly since the drug needed to traverse not only the lipid bilayer but also the three-dimensional network structure of the gels. However, in the same formulation, when changing the concentration ratio of P407 and P188, it could be seen that blank gels at 32 °C had the highest drug release in a certain time. For PAE-L-G, the final R% of the three different temperatures were 41.76 ± 3.78%, 46.14 ± 1.04%, and 43.47 ± 0.68%, respectively. There is no significant difference. In summary, the thermosensitive gels not only played the function of drug storage, but also had the ability to help drugs penetrate the skin, so they can be used as a carrier for local skin delivery. Meanwhile, it was further indicated that PAE-L-G at 32 °C was more suitable for the treatment of symptoms caused by AD.

At the same time, OriginPro 2021 software was used to simulate the release curve, and common models such as Zero-order kinetics, First-order kinetics, Higuchi, Korsmeyer–Peppas, Hixson–Crowell, and WeibullCDF were used to analyze the drug release kinetics. Among them, Table 2 lists the equations of the fitting curve whose correlation coefficients are greater than 0.95 and the worst fitting effect, and also shows the corresponding kinetic constants and exponential parameters. The WeibullCDF model can make R^2^ > 0.97, indicating that it is more suitable for this model and has feasibility and a linear relationship, while the Hixson–Crowell model has negative R², indicating that the time-cumulative drug release curve does not conform to the trend of this model.

### 2.5. Antioxidant Capacity via Scavenging Free Radicals

#### 2.5.1. Experiment of the Oxidation Resistance on H_2_O_2_

Hydrogen peroxide is a kind of reactive oxygen species (ROS). In the human body environment, it can be produced from many biological membranes, such as the mitochondrial membrane, microsomal membrane, and cytoplasm [53]. When the body’s internal REDOX balance system is broken, ROS accumulates too much, especially in skin cells, which can accelerate skin aging, induce a cellular inflammatory response, and inhibit the immune function of skin cells.

**Figure 2:**
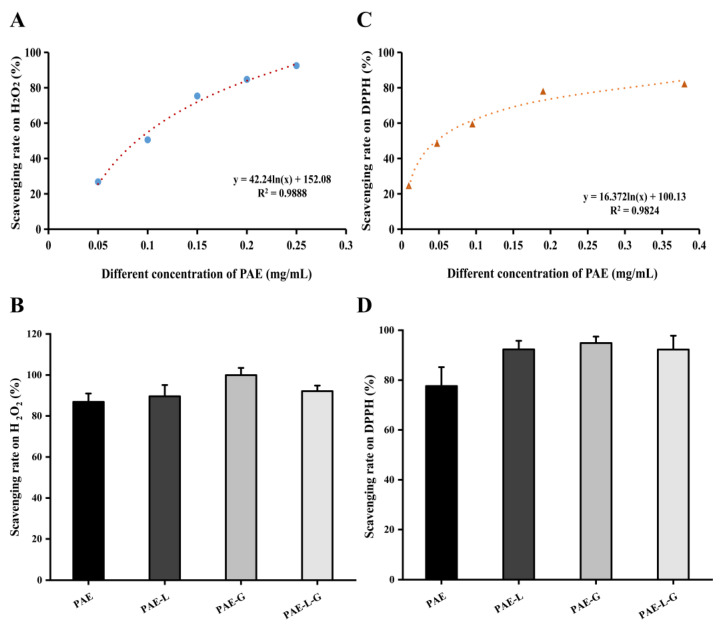
Free radical scavenging capacity. (**A**) Scavenging ability of paeonol at different concentrations on H_2_O_2_ free radicals. (**B**) Expression of H_2_O_2_ free radical scavenging ability of different preparations of PAE. (**C**) Scavenging rate on DPPH by different concentrations of PAE. (**D**) The scavenging ability of PAE, PAE-L, PAE-G, and PAE-L-G to DPPH free radicals, respectively.

According to the curve of inhibition rate changing with the concentration in Figure 2A, the antioxidant rate was positively correlated with the concentration of PAE at five different concentrations (0.05, 0.1, 0.15, 0.2, and 0.25 mg/mL). The relationship between the drug concentration and the antioxidant rate was in line with the logarithmic curve, and the free radical inhibition rate showed an upward trend under the change in paeonol concentration, gradually approaching 100%. In Figure 2B, the inhibition rates of PAE, PAE-L, PAE-G, and PAE-L-G on free radicals were 86.89 ± 4.07%, 89.61 ± 5.49%, 99.49 ± 3.49%, and 92.12 ± 2.71%. At the same concentration, the free radical scavenging rate of PAE should be the maximum, because its release and reaction were direct, so it could have a better effect. However, in the experimental results, the inhibition rate of PAE-G was the best, possibly because poloxamer itself could be used as a solvent, increasing the surface solubility of paeonol. In addition, it had been proved that poloxamer 188 could protect cell membranes by eliminating free radicals [54]. Although paeonol in the PAE-L-G was coated with the lipid layer of liposomes and the triblock copolymer of gels, it still had a good antioxidant capacity due to the synergism of drugs and excipients of liposomes and gels.

#### 2.5.2. Scavenging DPPH Free Radical Effect

The DPPH radical is a synthetic, single-electron, stable, nitrogen-centered paramagnetic compound. When the free radical scavenger is present, DPPH accepts an electron or hydrogen atom to form a stable DPPH-H compound, which will fade the solution and reduce absorbance. The degree of discoloration is quantitatively related to the number of electrons received (free radical scavenger activity) [55,56]. Due to the simple structure of DPPH free radicals and the easy control of reaction, DPPH free radicals can be used as an evaluation index of antioxidant performance.

During the experiment, shown as Figure 2C,D, the scavenging rate on free radicals increased with the difference in drug concentration and reaction time with DPPH. Because the scavenging rate of free radicals except PAE can reach more than 90% within 2 h of each prescription, the experimental results showed the same trend as the H_2_O_2_ experiment, and the effect of PAE-G was the best, mainly because poloxamer gels played a synergistic role in this reaction process. The results showed that the PAE-L-G could fully exert the scavenging free radical effect. There was no significant difference between the groups.

### 2.6. Pharmacodynamic Studies of Atopic Dermatitis

#### 2.6.1. Reduce AD-like Skin Symptoms

**Figure 3:**
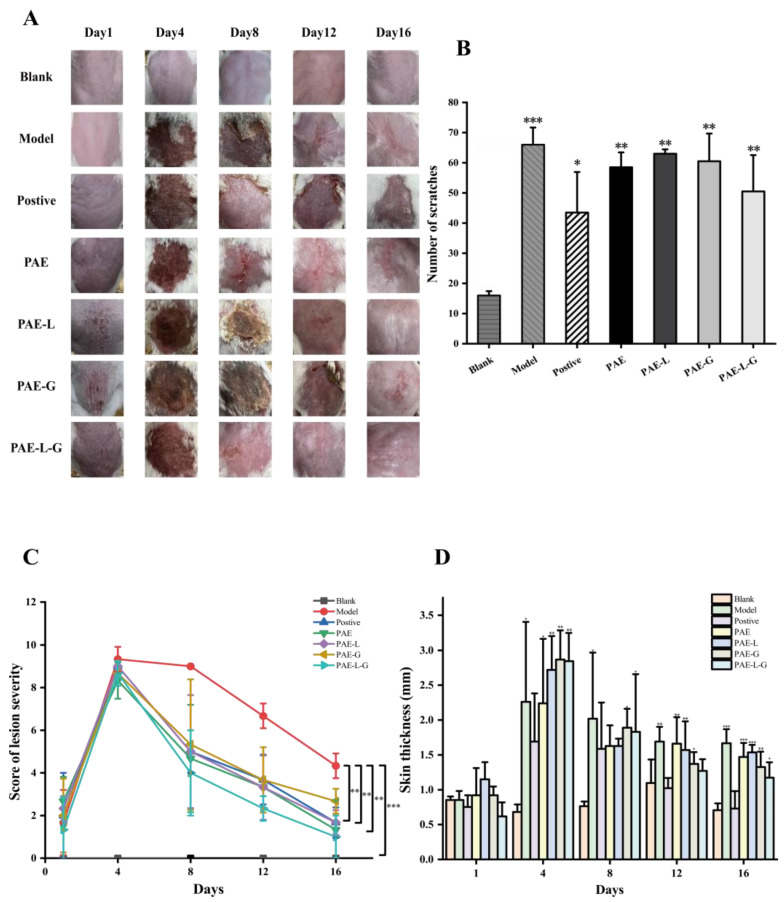
Skin conditions and behavioral manifestations in AD-like mice. (**A**) Skin surface symptoms of mice. (**B**) Number of scratches. (**C**) Score of lesion severity. (**D**) Skin thickness. This difference is statistically significant. (*** *p* < 0.001; ** *p* < 0.01 and * *p* < 0.05 vs. normal group).

The atopic dermatitis model was performed on the skin of mice except for the blank group in the first four days. As can be seen from Figure 3A, no obvious symptoms of skin injury were observed on the first day of DNCB administration. It can be seen in Figure 3C,D that the skin thickness and injury severity of each group reached a peak on the fourth day. Therefore, dry skin and peeling symptoms began at five days. The secondary sensitization on the ear was performed on the 8th, 12th, and 16th days, respectively, but the corresponding skin symptoms had been alleviated to some extent. The skin status of the positive control group and PAE-L-G group was restored to 70% on the 8th day; there were still scabs and skin damage in PAE, PAE-L, and PAE-G groups, and only the skin of the PAE-L-G group recovered on the last day. As scratching times were recorded on the last day, Figure 3B showed that *p* values between the administration group and the model group were all greater than 0.05, with no significant difference. In this experiment, compound dexamethasone acetate cream was used as a positive control, but it was found that the mice showed emaciation and slow skin recovery after the 8th day, which even led to the death of the mice. The main reason was that dexamethasone belongs to the hormone class, which can not only achieve anti-inflammatory, anti-sensitization, antipruritic effects, as well as reduce exudation, but it can also lead to skin atrophy and fungal infection, causing physical damage. Therefore, it is necessary to find more effective alternatives.

The spleen is an important immune organ of the body, which plays a role in indicating the strength of immune function [57]. The spleen index of AD-like mice induced by DNCB is shown in Table 3. The spleen in the model group accounted for the largest proportion, indicating spleen enlargement caused by inflammatory symptoms of atopic dermatitis. In terms of the experimental results, the degree of spleen enlargement in the PAE-L group was slightly lower than that in the model group, and the group with temperature-sensitive gel could achieve the same effect as that in the blank group, expressing that the addition of gel could reduce the body’s immunity and inhibit dermatitis. The difference was not statistically significant (*p* > 0.05). As the ear was sensitized three times, but the ear was not treated with drugs, the measurement of ear swelling degree on the 17th day showed that, compared with the model group, PAE-L-G could better inhibit the inflammation damage, hypertrophy, and swelling of the ear, and even showed better efficacy than the positive control group, and that the inhibition rate of paeonol solution was lower. Combined with these results, PAE-L-G could be effective not only for topical use but also for systemic inflammation.

#### 2.6.2. PAE Content in the Skin of AD-like Mice

**Figure 4:**
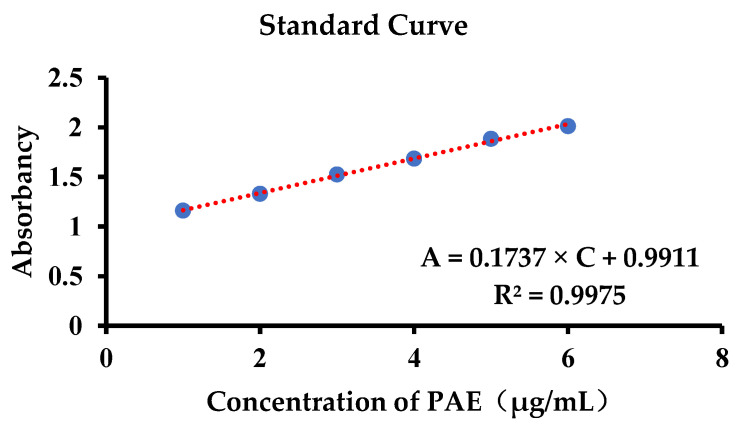
Standard curve of PAE in the skin homogenate.

To detect the drug content in the skin of AD experimental mice, the standard curve of skin homogenate (Figure 4) was first established according to the concentration of the standard curve established previously. The results show that: A = 0.1737 × C + 0.9911 (R^2^ = 0.9975), and the absorbance has a linear relationship with this concentration range. According to the measurement of mice in the administration group (Table 4), the drug content in the PAE group was the highest, up to 1.89 ± 0.59 μg/mL, while that of PAE-L-G was slightly higher than that of the PAE-L and PAE-G groups.

#### 2.6.3. Inhibit the Production of MDA in Tissue Homogenate

Oxygen free radicals act on the unsaturated fatty acids of lipids to produce lipid peroxide. The latter is gradually decomposed into a series of compounds, including malondialdehyde (MDA). Lipid oxidation levels can be detected by detecting MDA levels. Under acidic and high temperature conditions, MDA can be condensed with thiobarbituric acid (TBA) to produce brown–red trimethylecylate, which can be detected at 532 nm. Studies have shown that the detection of MDA levels could be used as a test index for the symptoms of photoaged skin caused by ultraviolet exposure [58]. Fardin [59] used MDA as an antioxidant parameter to study oxidative stress degree in atopic dermatitis.

**Figure 5:**
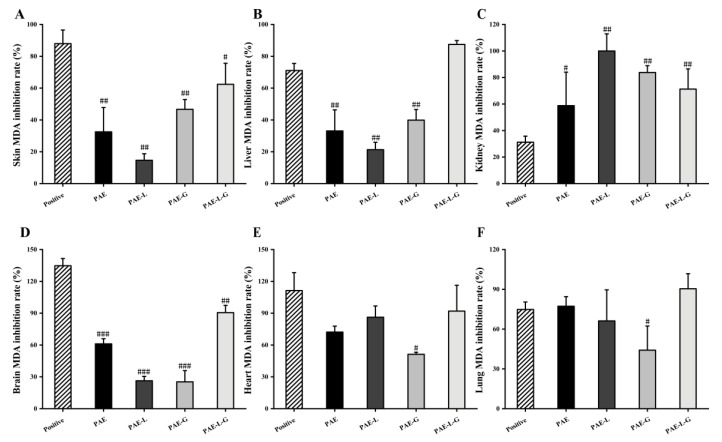
Inhibitory rate of different samples on MDA production in different organ homogenates. (**A**) Skin; (**B**) Liver; (**C**) Brain; (**D**) Kidney; (**E**) Heart; (**F**) Lung. The data are means ± SD (n = 3). ^###^ *p* < 0.001; ^##^ *p* < 0.01 and ^#^ *p* < 0.05 vs. control group.

In the AD experiment, MDA levels were detected in all organs of mice (Figure 5). There was no significant difference between PAE-L-G and positive control in the supernatant of organ tissue homogenate, indicating that PAE-L-G had a high inhibitory ability of lipid peroxidation in AD mice and could well inhibit oxidative stress response. The inhibition rates of PAE-L-G in the skin, liver, and brain were 50.03 ± 13.25%, 89.15 ± 2.43%, and 95.38 ± 6.97%, respectively, while those in the positive control were 93.99 ± 8.50%, 74.19 ± 4.46%, and 139.54 ± 6.79%. In the heart, lung, and kidney, PAE-L-G had no statistical significance compared to PAE, PAE-L, and PAE-G (*p* > 0.05).

#### 2.6.4. Blocking the Damage of AD-like Mouse Skin

Since AD is the primary manifestation of type 2 inflammatory disorder in keratinocytes, it leads to the increase in reactive oxygen species (ROS) in the cells [60,61]. The skin is exposed to the environment, oxygen, and bacteria for a long time, which induces oxidative stress response. ROS is produced by the immune system and has a good defense effect, but when ROS levels are too high in the body, the number of genes expressing inflammatory cells increases, leading to the persistence and deterioration of the inflammatory reaction. In order to prevent the loss caused by ROS, antioxidant enzymes such as superoxide dismutase (SOD), catalase (CAT), and glutathione peroxidase (GPx) are released as a protective mechanism [62]. SOD has the function of dismutating two superoxide radicals into hydrogen peroxide and oxygen. Thus, it has anti-aging and antioxidant effects on the skin, which can be used as an indicator to detect AD-like symptoms.

**Figure 6:**
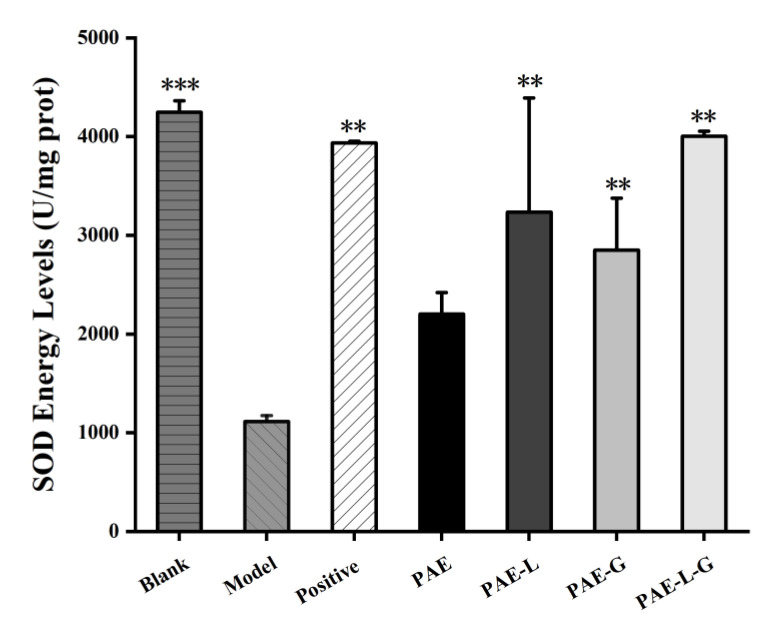
SOD activity value of mice skin. Values represent mean ± SD (n = 3, standard one-way ANOVA, *** *p* < 0.001 and ** *p* < 0.01 vs. model group).

In Figure 6, except for the PAE solution, SOD activity content in other drug administration groups was significantly higher than that in the model group (*p* < 0.01), showing statistical significance. Among them, PAE-L-G could achieve the same effect as the positive control, and could even make SOD activity reach a non-oxidation level. Combined with the results, PAE-L-G could relieve inflammatory symptoms caused by ROS-induced oxidative stress and it played an antioxidant role.

Due to the limited clinical application of paeonol, the encapsulation substrates are currently divided into conventional dosage forms, polymer delivery systems, and lipid-based delivery systems [63]. Tablets are common dosage forms on the market, which are used for oral administration, and the quality is stabilized through the coating technology. In Guo group’s study [64], paeonol and gastroretention tablets of paeonol were applied to gastric ulcer models at the same time. Changes in dosage forms can increase the residence time of drugs in the stomach and give full play to the advantages of local administration. However, rapid drug absorption and first-pass elimination effects necessary for oral administration still exist, reducing the bioavailability of the drug. The transethosomes in the lipid system show the ability to deform into cellular vesicles. The fluidization of stratum corneum (SC) lipid is induced by adding ethanol, which is conducive to drug penetration through the skin barrier and increases drug penetration. The application of paeonol transethosome to pig ear skin improved the stability of the drug and skin deposition [65]. The bulk of the preparations in polymer delivery systems consists of micron and nanoscale particles, such as microparticles, nanocapsules, and nanospheres, which can improve drug efficacy, safety, and controllability and reduce toxic side effects. At the same time, it has great potential for application in skin diseases. However, the preparation process is complicated and the research cost is high. In our study, by preparing paeonol-loaded liposomes in heat-sensitive gels, on the one hand, the intelligent responsiveness improved its targeting; on the other hand, diffusion experiment can find that the liposome gel system enhances its continuous drug release time, and the local drug concentration increases. The detection of antioxidant properties reflects the observable treatment for AD-like immune skin diseases.

## 3. Conclusions

In this study, PAE-L-G suitable for the treatment of AD was prepared through film dispersion and cold expansion, and its characterization and physicochemical properties were evaluated, which proved that its platform based on transdermal drug delivery was feasible. The results of in vitro diffusion experiments proved that, compared to the PAE solution, PAE-L-G had a better ability of controlled release and sustained efficacy for a longer time. In addition, different mathematical models were used to fit the release curve, which conforms to the first-order release kinetics and WeibullCDF. Free radical scavenging studies showed that liposomes and the network structure of gels might affect drug release. The inhibition rate of PAE-L-G with antioxidant properties was lower than that of other preparation groups, but there was no significant difference. Meanwhile, MDA experiments on various organs also proved that PAE-L-G could be used as an antioxidant. The results of behavioral and histological experiments on the AD mouse model indicated that PAE-L-G could relieve skin dryness, damage, and itching, even without the side effects of the positive control group, and so PAE-L-G could pass across the SC to work. T-SOD was used as an antioxidant enzyme; higher enzyme activity could be used as an index to evaluate the amount of ROS production, and PAE-L-G could reach a level similar to that of the blank group. The hydrogel itself can be used as the loading material of emollients. At the same time, the local therapy for AD can improve the skin condition, enhance the moisturizing ability, and can play a synergistic effect with paeonol. Poloxamer gels can respond to the change in character according to the change in temperature. The high antioxidant properties of paeonol can alleviate the symptoms of skin damage caused by immune reactions and reduce the generation of toxic side effects. In summary, we believe that paeonol-loaded liposomes in thermally reversible hydrogels are promising materials for skin delivery and have broad application prospects.

## 4. Materials and Methods

### 4.1. Materials and Reagents

Paeonol (purity 99%) and Hydrogen peroxide (H_2_O_2_) were obtained from Shanghai Suyi Chemical Reagent Co., Ltd. (Shanghai, China) and soybean phospholipids (purity 99%), cholesterol (purity 99%), NaOH, and Trichloroacetic acid (TCA) were purchased from Sinopharm Chemical Reagent Co., Ltd. (Shanghai, China), including sulfate heptahydrate. Poloxamer 407 and 188 were purchased from BAST Company. KH_2_PO_4_ was purchased from Guangxilong Science Co., Ltd. (Guangxi, China) and 1,1-diphenyl-2-picrylhydrazyl (DPPH, 98%), thiobarbituric acid (TBA), and 2,4-Dinitrochlorobenzene (DNCB) were provided by Shanghai Yuanye Biology Science and Technology Co., Ltd. (Shanghai, China). A T-SOD Test kit was acquired from Jiancheng Bioengineering Institute (Nanjing, China). All the reagents were AR grade.

### 4.2. Animals

Healthy Kunming mice (females weighing 20 ± 2 g) were purchased from the Animal Laboratory Center of Anhui University of Traditional Chinese Medicine (Hefei, China). The animal experiment protocol involved in this experiment conforms to the review and approval of the Ethics Committee of Anhui University of Chinese Medicine (Hefei, China). The indoor temperature was kept at 25 ± 2 °C, the relative humidity was kept at 50–70%, the light rhythm was 12 h, the feeding environment was quiet and ventilated, and the experimental animals were given ordinary feed and clean water.

### 4.3. Establishment of Standard Curves

The 0.0119 g of paeonol was accurately weighed with a balance, dissolved by adding a small amount of anhydrous ethanol, and then fixed into a 100 mL volumetric flask with phosphate buffer (PBS, pH 7.4). The mother liquor was completely dissolved by ultrasound to obtain 119 μg/mL. It was then diluted into 1.19 μg/mL, 3.57 μg/mL, 5.95 μg/mL, 8.33 μg/mL, 10.71 μg/mL, 13.09 μg/mL solution. Absorbance was measured at 274 nm with an ultraviolet spectrophotometer (UV-1000, AOE Instrument Co., Ltd., Shanghai, China). Values were recorded, and standard curves were established.

### 4.4. Preparation of Paeonol-Loaded Liposomes (PAE-L)

PAE-L was prepared using the thin-film dispersion method. Firstly, according to the ratio of phospholipid and cholesterol prescribed in our laboratory [58], 0.3 g phospholipid, 0.1 g cholesterol, and 0.0028 g paeonol were accurately weighed and placed into a 250 mL beaker, and then 5 mL anhydrous ethanol was added and placed into the ultrasonic cleaner (HS3120, Tianjin Hengao Technology Development Co., Ltd., Tianjin, China) to dissolve completely and quickly spread on the beaker wall rotary evaporator to form liposome film, and an appropriate amount of PBS was added to shake it with magnetic stirrers.

### 4.5. Characterization of PAE-L

The particle size and potential of PAE-L were measured by the Malvern nano-particle size potential analyzer (ZEN3690, US Malvern Instrument Co., Ltd., Malvern, UK) [66]. The encapsulation rate (ER%) of PAE-L was determined as follows: the 10 mL volumetric bottle was washed, dried, and set aside. A total of 1 mL PAE-L was placed in a 10 mL volumetric bottle, and PBS buffer was added for constant volume. The bottle stopper was pressed and mixed evenly. Then, 4 mL of the mixture was precisely absorbed, centrifuged at 4500 rpm for 15 min, and the supernatant was taken. The absorbance was measured at the maximum absorption wavelength of paeonol at 274 nm. According to the standard curve of paeonol drawn previously, the concentration of the drug that failed to be encapsulated by liposome was calculated. Next, 1 mL anhydrous ethanol was added to 1 mL PAE-L taken out again from a 10 mL volumetric bottle, ultrasonic dissolution was completed, and the concentration of all drugs in the supernatant was measured. The formula of drug encapsulation rate (ER%) is shown in (1):

(1) $$ER\%=\frac{\left[C_{w}-C_{n}\right]}{C_{w}}\times 100\%$$

where “*C_n_*” is the concentration of the drug that has not been successfully encapsulated and “*C_w_*” is the concentration of the drug encapsulated in the liposome.

### 4.6. Preparation of Paeonol-Loaded Liposomes in Thermoreversible Gels (PAE-L-G)

The prescribed amount of Poloxamer 407 and Poloxamer 188 were mixed and stirred evenly and the volume was fixed into the final preparation system and swelled overnight in a 4 °C refrigerator. PAE-L was evenly dispersed in the thermosensitive gels to finally prepare the PAE-L-G [67].

### 4.7. Characterization of PAE-L-G

#### 4.7.1. Visual Appearance and Clarity

We visually observed the appearance and transparency of formulations against a black background.

#### 4.7.2. Gelling Temperature and Gelling Time

The change in gel from a flowing solution state to a non-flowing solid state can be observed by the tube inversion method [68,69]. Therefore, the gel temperature of temperature-sensitive gel can be obtained by this method. We washed and dried multiple test tubes to avoid interference from impurities. A total of 2 mL of gel with different prescription concentrations were prepared and placed in a controlled, constant temperature, water bath environment. The temperature was adjusted at the rate of 0.1 °C per minute, and the gel was kept in it for 1 min. Then, the tube was vertically raised to 90° to detect whether the gel flowed. According to the ratio of different concentrations of P407 and P188, the temperature control range was set between 20 °C and 40 °C, and three parallel measurements were made to take the mean value.

Detection of gelation time is also called gelation capacity, and we measured this value using the same test tube method as the temperature measurement, with a stopwatch to record the time of hydrogel gelation, that is, the gelation capacity.

#### 4.7.3. Determination of Viscosity

**Figure 7:**
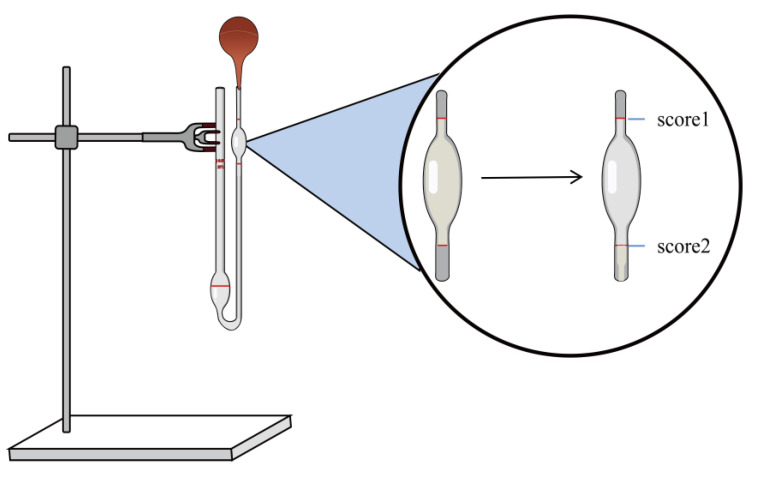
Method of measurement for viscometers.

The viscosity was determined using an Ostwald viscometer (1831, Ring Light Glass Instrument Co., Ltd., Taizhou, China) [68]. Before measurement, the Ostwald viscometer was cleaned with anhydrous alcohol, and then the alcohol was volatilized. We fixed the viscometer on an iron frame and kept the liquid level balanced (Figure 7). We then poured 5 mL of distilled water into the viscometer, use the ear wash ball to draw the liquid until it reached score 1, started timing, and stopped timing when the liquid level dropped to score 2.

Each group of samples was measured three times repeatedly, and the recorded decline time of the solution was calculated using the viscosity Formula (2), and, finally, the viscosity of the sample was obtained:

(2) $$\eta_{2}=\frac{\rho_{2}t_{2}}{\rho_{1}t_{1}}\times \eta_{1}$$

In the above formula, “η” represents viscosity (MPa·S), “ρ” represents density (g/mL), and “t ” represents the time (s). The digits “1” and “2” refer to water and sample, respectively.

#### 4.7.4. pH Value

The pH of the liposomes-in-gels was measured using a calibrated pH meter (PHS-3E, Shanghai Lei Instrument Co., Ltd., Shanghai, China). The samples were made in triplicate and the parallel measurements were repeated three times to calculate the mean value of the preparation.

### 4.8. Antioxidant Ability

#### 4.8.1. Scavenging H_2_O_2_ Assay

Hydrogen peroxide solution was prepared by dissolving 0.1 mL hydrogen peroxide in 50 mL PBS. The samples of the three groups were prepared at the same time. The blank group was treated with 0.6 mL PBS and 1.8 mL H_2_O_2_ solution, and the sample group was treated with 0.6 mL different preparations and 1.8 mL H_2_O_2_ solution, and the mixed solution of 0.6 mL different preparations and 1.8 mL PBS was used as the control group. After preparation, the three groups of preparations were incubated at room temperature for 10 min and protected from light, and the absorbance was measured at the detection wavelength (230 nm), and the clearance rate was obtained according to Formula (3):

(3) $$\mathrm{RSA}\;\mathrm{of}\;\mathrm{antioxidant}\;\mathrm{activity}=\left[1-\frac{A_{S}-A_{X}}{A_{O}}\right]\times 100\%$$

where “$A_{S}$” represents absorbance of the sample group, “$A_{X}$” represents absorbance of the control group, and “$A_{O}$” represents absorbance of the blank group.

#### 4.8.2. Scavenging DPPH Free Radical

A total of 8 mg of DPPH (1,1-diphenyl-2-trinitrophenylhydrazine) was weighed into a 100 mL weighing bottle with ethanol absolute. The experiment was divided into the blank group, sample group, and control group. The blank group was mixed with 1 mL PBS and 2 mL DPPH solution, the sample group was mixed with 1 mL of different preparations prepared and 2 mL DPPH solution, and the control group was mixed with 2 mL PBS and 1 mL preparation. After being mixed evenly and gently shaken, they were allowed to react for 2 h in the environment while avoiding light. Finally, the absorbance was measured at 517 nm, and the result was obtained by using the clearance Formula (3).

### 4.9. In Vitro Drug Release across the Dialysis Membrane

The improved Franz diffusion well was adopted [70], with a diffusion area of 1.825 cm^2^ and the receiving cell volume was 15 mL. We prepared three sets of controllable temperature magnetic stirrers (85-2, Changzhou Yineng Experimental Instrument Factory, Changzhou, China), and set the temperatures to 32 °C, 35 °C, and 37 °C, respectively, which were stable between ±0.5 °C. Samples of different gelling temperatures were placed on it, while PBS buffers (pH = 7.4) were prepared as a supplement to the receiving pool. The same volume of PBS was added for every 2 mL of sample solution collected. 5 min, 10 min, 20 min, 30 min, 1 h, 2 h, 3 h, 4 h, 5 h, 6 h, 7 h, 8 h, 9 h, 10 h, 11 h, 12 h, 24 h, 36 h, 48 h, 60 h, 72 h, 84 h, and 96 h were used as sampling nodes, and the absorbance of samples was timely measured at 274 nm. This step was repeated three times in parallel, and the cumulative drug release rate *Q*_*n*_ (%) was obtained according to Formula (4):

(4) $$Q_{n}\left(\%\right)=\frac{\left(C_{n}\times V_{n}+\sum_{i=1}^{n-1}C_{i}\times V_{i}\right)}{Q_{T}}\times 100\%$$

where “$C_{n}$” is the concentration of the drug sampled each time, “$V_{n}$” is the volume of dissolved liquid sampled each time, “$C_{i}$“ and “$V_{i}$” are the accumulated concentration and volume of the drug sampled before, and “*Q_T_*” is the theoretical drug amount.

The function of the fitting curve is to use a mathematical model to fit a series of data into a smooth curve, explore the internal relationship between the two groups of data, and understand the change trend between the data. Therefore, in order to study the drug release mechanism of each sample group, a variety of fitting models were selected to determine the best fitting curve.

### 4.10. Preliminary Antioxidant Activity in Atopic Dermatitis

#### 4.10.1. Establishment of the Atopic Dermatitis Model

The mice were fed adaptively for several days, and the hair in a 2 × 2 cm² area was shaved on the back of the mice on the day before the start of the experiment. 5% DNCB solution was prepared (dissolved in a mixture according to the ratio of acetone: olive oil = 3:1), and 0.5% DNCB was diluted on a 5% basis. 100 µL of 5% DNCB solution was used to sensitize the back skin for 2 days. The blank group was given the same volume of acetone and olive oil carrier. After 5 days of intervals, the 0.5% DNCB solution was applied to ear skin 3 times, once every 3 days to sensitize. On the fourth day of the experiment, the blank group and the model group were treated with PBS, the positive control group was smeared with the same volume of compound dexamethasone acetate cream, and the administration group was treated with PAE, PAE-L, PAE-G, and PAE-L-G, respectively, for 12 consecutive days. The mice were fasted for 12 h after the final treatment and sacrificed on the 17th day of the experiment. During the experiment, the skin thickness of the mice’s backs and ears were collected for measurement. Organs were used for follow-up experiments. Spleen index was calculated by the ratio of the spleen to body weight, and the ear swelling degree was calculated by weight reduction of the left and right ears. Ear swelling inhibition rate was also recorded using Formula (5):

(5) $$Swelling\;inhibition\;rate\left(\%\right)=\frac{A_{m}-A_{s}}{A_{m}}\times 100\%$$

where “$A_{m}$” is the ear swelling degree of the model group and “$A_{s}$” is the ear swelling degree of sample groups.

#### 4.10.2. Evaluation of the Severity of Dermatitis and Observation of Scratching

Scratching behavior was recorded for 20 min after the last sensitization with 0.5% DNCB (on day 16). A mouse raised its paw and continued to scratch for a long time until it returned to the floor. This was recorded one time.

#### 4.10.3. Scoring of Dermatitis

The skin lesion score was mainly divided into four aspects: (I) skin flaring and hemorrhage, (II) crust formation and xerosis cutis, (III) edema, and (IV) excoriation and erosion, which were scored on three scales: 0 = no symptoms, 1 = mild, 2 = moderate, and 3 = severe. Finally, the total score of the four groups was used as the dermatitis score.

#### 4.10.4. Determination of Drug Content

The back skin of mice in the blank group was cut up and added to anhydrous ethanol (1:10 mL) to homogenate (FSH-2A, Changzhou Putian Experimental Instrument Factory, Changzhou, China) in three batches. The homogenate was wrapped with plastic wrap to prevent the volatilization of ethanol. Then, the skin was ultrasonic for 30 min and transferred to a centrifuge tube, and centrifuged at the speed of 3500 r/min for 5 min. Because the absorbance value of the supernatant measured directly was too large, the supernatant diluted 10 times with ethanol was selected. Six test tubes were added with 1mL of supernatant diluted 10 times, and then PBS and five drug concentrations were added, respectively. The *A* value was determined under the characteristic wavelength of PAE (274 nm), and the drug standard curve of skin homogenate was established. We then took 2 mL of the back skin homogenate supernatant of the drug administration group (diluted 10 times) to obtain the *A* value required by the absorbance-drug concentration equation and calculated the drug content.

#### 4.10.5. Inhibition of the Production of Malonedione (MDA)

The organs and skin tissues of the mice were removed on the 17th day after they were killed by neck removal. After repeated rinsing with physiological saline (pH = 7) to remove the residual blood, they were dried with filter paper. The tissue was weighed and 1:9 cold physiological saline was added for full homogenization (physiological saline was added three times), the samples were collected in test tubes, and the speed of the overspeed centrifuge (TG16-W, Hunan Xiangli Experimental Instrument Factory, Hunan, China) was set at 4000 rpm. After the time was kept for 15 min, the supernatant was taken. A total of 1 mL supernatant + 9 mL dilution medium was used to make 10% analytical solution.

After mixing 0.375% TBA and 5.6% TCA at a ratio of 2:1, 3 mL was added into 1 mL tissue homogenate. The thermostatic water bath was heated to 95 °C in advance, and the mixture was incubated in the test tube for 40 min. After the water cooled to room temperature and centrifuge spun at 4000 r/min for 8 min, we measured the absorbance value of the supernatant at wavelength 532 nm and calculated the inhibition rate (IR%):

(6) $$IR\left(\%\right)=\frac{A_{m}-A_{s}}{A_{m}-A_{b}}\times 100\%$$

where “*A*” is the absorbance value, “*m*” is the model group, “*s*” is the sample group, and “*b*” is the blank group.

#### 4.10.6. Evaluate the Antioxidant Effect of Superoxide Dismutase (SOD) in AD Mice Skin

The animal tissue weight was accurately weighed by adding a homogenate medium (0.9% physiological saline) of 9 times the volume at the ratio of weight (g): volume (mL) = 1:9. The homogenate was prepared into 10% homogenate using mechanical homogenization under the conditions of an ice water bath. The supernatant was taken for determination after being centrifuged at the speed of 2500–3000 rpm for 10 min. We then added the application solution of color removal agent into the monitoring and measuring tube and kept a constant temperature (37 °C) water bath for 40 min. Next, we added color-developing agent and mixed well, left the mixture at room temperature for 10 min, and, at the wavelength of 550 nm in zero distilled water, performed color comparison. Specific operations were conducted according to the experimental procedure of the SOD kit.

### 4.11. Statistical Analysis

All experiments in this paper were repeated three times, and the results were expressed by mean ± SEM. Data analysis charts were drawn by OriginPro 2021 (OriginLab Corp., Northampton, MA, USA) and statistical results were analyzed by one-way analysis of variance (ANOVA) with SPSS software (IBM, Armonk, NY, USA). A *p* value less than 0.05 was considered to be statistically different between the two groups of data.

## Figures and Tables

**Table 1:** Physicochemical properties of poloxamer gels at different temperatures.

	Group	Gelation Temperature (°C)	Gelation Capacity (s)	Viscosity 4 °C (MPa·S)	Viscosity 32–37 °C (MPa·S)	pH 4 °C	pH 32–37 °C
32 °C	G	31.25 ± 0.78	44.51 ± 2.60	19.32 ± 1.44	91.99 ± 0.65	7.25 ± 0.03	7.04 ± 0.02
	PAE-G	31.80 ± 0.42	44.79 ± 2.46	19.06 ± 0.55	92.80 ± 0.09	7.38 ± 0.01	7.16 ± 0.05
	PAE-L-G	31.70 ± 0.42	51.80 ± 5.05	30.34 ± 0.06	136.98 ± 0.78	7.33 ± 0.04	7.18 ± 0.03
35 °C	G	34.45 ± 0.49	58.29 ± 5.85	28.73 ± 0.39	94.44 ± 0.13	7.27 ± 0.02	7.11 ± 0.12
	PAE-G	34.70 ± 0.14	59.61 ± 0.86	29.58 ± 0.02	94.38 ± 0.51	7.48 ± 0.02	7.23 ± 0.27
	PAE-L-G	34.60 ± 0.57	62.92 ± 7.67	36.36 ± 6.32	149.28 ± 0.22	7.47 ± 0.01	7.23 ± 0.01
37 °C	G	36.90 ± 0.14	73.41 ± 1.35	50.07 ± 0.22	120.94 ± 1.41	7.47 ± 0.05	7.27 ± 0.17
	PAE-G	36.70 ± 0.28	69.66 ± 0.95	46.22 ± 7.13	123.43 ± 1.69	7.55 ± 0.06	7.34 ± 0.03
	PAE-L-G	36.70 ± 0.71	82.58 ± 2.34	56.39 ± 5.76	197.80 ± 7.14	7.52 ± 0.03	7.38 ± 0.14

**Table 2:** The fitting curve values under different mathematical models.

Group		Relevant Data	First-Order	WeibullCDF	Hixson–Crowell
PAE		Equation	Q = 74.1414 [1 – exp (−0.0152t)]	Q = 10.3580 + 63.8181 [1 − e^−(t/94.2344)^1.3040^]	Q = 100 [1 − (1 − 2.7546t)^3^]
		R²	0.9931	0.9984	−3.9777
PAE-L		Equation	Q = 55.2900 [1 – exp (−0.0017t)]	Q = −1.1750 + 5.2263 [1 − e^−(t/881.6552)^0.9298^]	Q = 100 [1 − (1 − 8.7042t)^3^]
		R²	0.9983	0.9988	0.6961
PAE-G	32 °C	Equation	Q = 57.0866 [1 – exp (−0.0020t)]	Q = 0.7776 + 56.4113 [1 − e^−(t/515.9190)^0.9777^]	Q = 100 [1 − (1 − 1.9744t)^3^]
		R²	0.9957	0.9961	−0.1147
	35 °C	Equation	Q = 49.7029 [1 – exp (−0.0013t)]	Q = 0.0281 + 50.8791 [1 − e^−(t/864.5301)^0.8868^]	Q = 100 [1 − (1 − 6.6639t)^3^]
		R²	0.9978	0.9995	0.6268
	37 °C	Equation	Q = 48.6397 [1 – exp (−0.0014t)]	Q = −0.6052 + 48.9826 [1 − e^(t/646.9570)^1.0363^]	Q = 100 [1 − (1 − 5.6325t)^3^]
		R²	0.9973	0.9996	0.6506
PAE-L-G	32 °C	Equation	Q = 40.6142 [1 – exp (−8.0875t)]	Q = −1.0828 + 41.8662 [1 − e^(t/1209.4106)^0.9575^]	Q = 100 [1 − (1 − 4.1847t)^3^]
		R²	0.9962	0.9971	0.6384
	35 °C	Equation	Q = 43.0370 [1 – exp (−0.0016t)]	Q = 1.3481 + 39.6793 [1 − e^−(t/565.3188)^2.0324^]	Q = 100 [1 − (1 − 5.6873t)^3^]
		R²	0.9574	0.9791	−0.0549
	37 °C	Equation	Q = 42.4447 [1 – exp (−0.0015t)]	Q = −1.7778 + 44.5795 [1 − e^−(t/662.4938)^0.9005^]	Q = 100 [1 − (1 − 5.4228t)^3^]
		R²	0.9930	0.9946	0.0349

**Table 3:** Spleen index and ear swelling. The data are means ± SD (n = 3). Means with different letters (a,b) are significantly different (*p* < 0.05) via Duncan’s test.

Group	Weight (g)	Splenic Weight (g)	Spleen Index (%)	Swelling Degree (mg)	Swelling Inhibition Rate (%)
Blank	28.14 ± 1.50	0.1077 ± 0.0187	0.38 ± 0.05	0.57 ± 0.64 ^b^	83.17
Model	21.31 ± 5.55	0.0921 ± 0.0096	0.46 ± 0.18	3.37 ± 0.70 ^a^	-
Positive	22.85 ± 1.57	0.0852 ± 0.0694	0.34 ± 0.07	1.25 ± 1.63 ^ab^	62.87
PAE	30.29 ± 2.82	0.1161 ± 0.0034	0.39 ± 0.05	2.50 ± 2.12 ^ab^	25.74
PAE-L	27.69 ± 0.97	0.1184 ± 0.0430	0.43 ± 0.17	2.90 ± 0.95 ^ab^	13.86
PAE-G	27.82 ± 2.18	0.1034 ± 0.0325	0.37 ± 0.09	2.47 ± 1.25 ^ab^	26.73
PAE-L-G	31.52 ± 1.17	0.1151 ± 0.0009	0.37 ± 0.01	1.23 ± 1.23 ^ab^	63.37

**Table 4:** Drug content of skin in different groups of AD-like mice.

Group	Drug Concentration (μg/mL)
PAE	1.89 ± 0.59
PAE-L	1.11 ± 0.33
PAE-G	1.15 ± 0.36
PAE-L-G	1.50 ± 0.41

## Data Availability

Not applicable.
